# Potential Protective Effect of* Achillea fragrantissima* against Adriamycin-Induced Cardiotoxicity in Rats via an Antioxidant and Anti-Inflammatory Pathway

**DOI:** 10.1155/2019/5269074

**Published:** 2019-06-17

**Authors:** Maha A. Hijazi, Hanan A. Jambi, Buthaina M. Aljehany, Maha A. Althaiban

**Affiliations:** Food and Nutrition Department, Faculty of Home Economics, King Abdulaziz University, Jeddah, Saudi Arabia

## Abstract

Adriamycin (Adr) is a cytotoxic anthracycline agent that is utilized to manage many types of tumors, but its clinical use is undesirable due to severe cardiotoxicity. The present study aimed to investigate the cardioprotective effect of* Achillea fragrantissima* (*A. fragrantissima*) against Adr-induced cardiotoxicity through the antioxidant and anti-inflammatory metabolic pathways. A single dose of Adr was injected in rats to induce cardiotoxicity. Rats are divided into 5 groups, control,* A. fragrantissima* 800, Adr,* A. fragrantissima* 400 + Adr, and* A. fragrantissima* 800 + Adr. 72 h after Adr administration, electrocardiographic (ECG) study was performed for all rats. Serum and hearts were then collected for biochemical and histopathological studies.* A. fragrantissima* ameliorated Adr-induced ST-segment elevation. It reduced Adr-induced elevation in lactate dehydrogenase (LDH), creatine kinase-MB (CK-MB), thiobarbituric acid reactive substance (TBARS), tumor necrosis factor-alpha (TNF-*α*), interleukin-1 beta (IL-1*β*), and IL-6. It also protected against Adr-induced histopathological changes. Pretreatment with the extract increased heart tissue contents of glutathione peroxidase (GSH-PX) and reduced glutathione (GSH). Phytochemical analysis of the extract revealed that it is rich in phenolic and flavonoid active constituents. The results of this study revealed that* A. fragrantissima* extract ameliorates Adr-induced cardiotoxicity* via* an antioxidant and anti-inflammatory mechanisms. Further studies are warranted in order to recognize the precise active constituents of this natural extract which are responsible for the antioxidant and anti-inflammatory actions.

## 1. Introduction

Adriamycin (Adr) is a cytotoxic anthracycline antibiotic that is used to treat a wide variety of cancers including, leukaemia and lymphoma, besides breast, lung, and other solid cancers. However, its clinical application is restricted due to its dangerous cardiotoxicity that may be aggravated to heart failure [1–3]. The underlying mechanism of Adr-induced acute cardiotoxicity is based on the production of free radicals and reactive oxygen species (ROS) that damage the cell membrane lipids and caused the liberation of lipid peroxides products [4, 5]. Moreover, there is growing evidence that Adr also elicits inflammatory effects in the vasculature and the myocardium that subsequently induces the production of several proinflammatory mediators such as tumor necrosis factor-alpha (TNF-*α*) [6]. Interleukin-1 beta (IL-1*β*) is an initiator cytokine that exhibits a great function as a regulator of the immunoinflammatory pathway. IL-1*β* has been disclosed to contribute to the Adr-induced rises in the levels of IL-6 and perform a very distinct role in Adr-induced cardiotoxicity [7]. Research has found that cardiac toxicity of Adr is also associated with an increase in IL-1*β* production. Il-1B causes cardiomyocytes apoptosis by increasing the calcium storage of the heart muscle cells [8].

Recently, many studies documented that natural products rich in antioxidants have an effective role in the prevention of cardiac toxicity of Adr [9]. The preventive mechanism for natural remedies against Adr-induced cardiotoxicity is still unclear. However, research has shown that the mechanism may be limited to the elimination of ROS or prevention against the harmful cardiovascular effects of the metabolic syndrome [10–12].* A. fragrantissima* is one of the desert herbs belonging to the family Asteraceae. Historically, the herb is used in the Arab region as an alternative treatment for diabetes, gastrointestinal diseases, and respiratory diseases [13–16].* A. fragrantissima* contains a high percentage of phenolic and flavonoid active constituents such as achillolide A, swertisin 2′′-arabinosideafroside, cirsimaritin, chrysoplenol, cirsiliol, eupatilin-7-methyl ether, and isovitexin 4′- methyl ether [17–19]. Sesquiterpene lactones are a group of active substances, which have shown a great biological efficacy as anti-inflammatory, antioxidant, and neuroprotective compounds [20–23]. Achillolide A, a sesquiterpene lactone, is one of the most effective substances separated from many of the Asteraceae family, which has shown a strong anti-inflammatory effect and sweeping ROS and also prevents the formation of nitric oxide (NO) [18].

Since oxidative stress and inflammation have become accepted as a suitable target for early therapeutic intervention in Adr-induced cardiotoxicity, the present study addressed the cardioprotective effect of* A. fragrantissima* against Adr-induced cardiotoxicity through the antioxidant and anti-inflammatory metabolic pathways.

## 2. Methods

### 2.1. Drugs

Drugs included are Adr (Adriamycin®) 50 mg/25 ml, EBEWE Pharma, Austria, Urethane (Sigma Aldrich Inc., USA).

### 2.2. Collection of* A. fragrantissima*

The plant was collected from Al-Jawf in Northern Saudi Arabia. The taxonomic identification of the plant was confirmed by botanists in Pharmaceutical and Phytochemistry Department, Faculty of Pharmacy, KAU, Jeddah, Saudi Arabia.

### 2.3. Preparation of Plant Extract

The aerial parts of* A. fragrantissima* were dried at 40°C and grinded. 500 g of* A. fragrantissima* powder was then extracted with methanol and water mixture (80%). The final collected extract was filtrated and concentrated to dryness under reduce pressure at < 35°C using a rotary evaporator (Rota vapor R-215, B$\overset{-}{\ddot{u}}$tchi, Switzerland). Finally, the dried extract was transferred to a Gamma 2-20 freeze dryer (Christ, Osterode i. H., Germany) for 48 h, at – 20°C to yield a solid extract (100 g yield 4.27 g), and then stored at 4°C until further use. 100 mg of dried* A. fragrantissima* extract was dissolved in 200 ml distilled water to yield a solution with a final concentration of 0.5 mg/ml, centrifuged to remove any undissolved component [24].

### 2.4. Phytochemical Analysis

The phytochemical screening of* A. fragrantissima* extract has been performed to find the presence of the major chemical constituents, including alkaloids, flavonoids, glycosides, saponins, tannins, resins, and triterpenoids using standard procedures of analysis [25].

### 2.5. Experimental Design

In this study, 50 adult male Wister rats (180-200 g body weight) were divided into 5 groups (n = 10). The rats were obtained from King Fahd medical research center, KAU, Jeddah, Saudi Arabia. They handled according to the roles and rights of animal research, KAU. The rats housed at 22 ± 3°C and maintained free axis to food and water. The study groups were as follows: Group (I) (control), the rats injected with normal saline; Group (II) (*A. fragrantissima *800), the rats orally injected with* A. fragrantissima* extract at a dose of 800 mg/kg/day; Group (III) (Adr), the rats injected i.p. with Adr (10 mg/kg) [26]; Group (IV) (*A. fragrantissima *400 + Adr), the rats orally injected with* A. fragrantissima* extract at a dose of 400 mg/kg/day for 2 weeks [27] and then injected with Adr (10 mg/kg); Group (V) (*A. fragrantissima *800 + Adr), the rats orally injected with* A. fragrantissima* extract at a dose of 800 mg/kg/day for 2 weeks and then injected with Adr (10 mg/kg).

### 2.6. Electrocardiography (ECG) Assessment

72 h after Adr injection, the PowerLab system (ADI Instruments) connected to a PC running LabChart professional software (version 7.3) containing an ECG module was used to conduct the standard limb lead II of the surface ECG for each rat separately [28].

### 2.7. Samples Collection

72 h after Adr injection, rats were anesthetized with ether, blood samples were collected, and serum was separated and kept frozen at -80°C for the biochemical determination of the cardiac enzymes and inflammatory cytokines (IL-1*β*, TNF-*α*, and IL-6). The rats were then decapitated, and the hearts were collected and maintained either frozen at -80°C for the ELIZA determination of thiobabituric acid reactive substances (TBARS), glutathione peroxidase (GSH-PX), and reduced glutathione (GSH) or in 10% buffered formalin solution for the histopathological study.

### 2.8. Determination of Serum Creatine Kinase-MB (CK-MB) and Lactate Dehydrogenase (LDH)

Serum CK-MB and LDH activity were determined using ELISA kits obtained from MyBiosource, San Diego, California, USA, according to the manufacture instructions.

### 2.9. Preparation of Heart Tissues Homogenate

The heart tissues were homogenized in PBS (1:9) using a Teflon pestle (Ultra-Turrax, IKA: T25 digital, Germany) and centrifuged at 12000 g for 15 min at 4°C (Centurion, K280 R, UK). The supernatants were used for the estimation of TBARS, GSH-PX, and GSH.

### 2.10. Determination of Heart Tissue TBARS, GSH-PX, and GSH Concentration

TBARS, GSH-PX, and GSH were determined using ELISA kits obtained from MyBiosource, San Diego, California, USA, according to the manufacture instructions.

### 2.11. Determination of Serum TNF-*α*, IL-1*β*, and IL-6 Concentration

TNF-*α*, IL-1*β*, and IL-6 were determined using ELISA kits obtained from MyBiosource, San Diego, California, USA, according to the manufacture instructions.

### 2.12. Histopathological Examination

The formaldehyde fixed hearts were paraffin-embedded, cut into sections, and then stained with Hematoxylin-Eosin (H & E). The slides were then examined microscopically.

### 2.13. Statistical Analysis

Results were presented as mean ± SE. The means were compared by one-way analysis of variance (ANOVA), followed by Tukey's HSD, to determine the statistical significance of the difference using SPSS version 22. P ≤ 0.05 indicate significance difference.

## 3. Results

### 3.1. Phytochemical Analysis of* A. fragrantissima* Extract

Phytochemical analysis of* A. fragrantissima *extract indicated that it contains adequate amounts of flavonoids, phenolics, glycosides, lignans, and terpenes (Table 1).

### 3.2. Effects of* A. fragrantissima* on Adr-Induced Changes in ECG Tracing Pattern and Parameters

**Figure 1:**
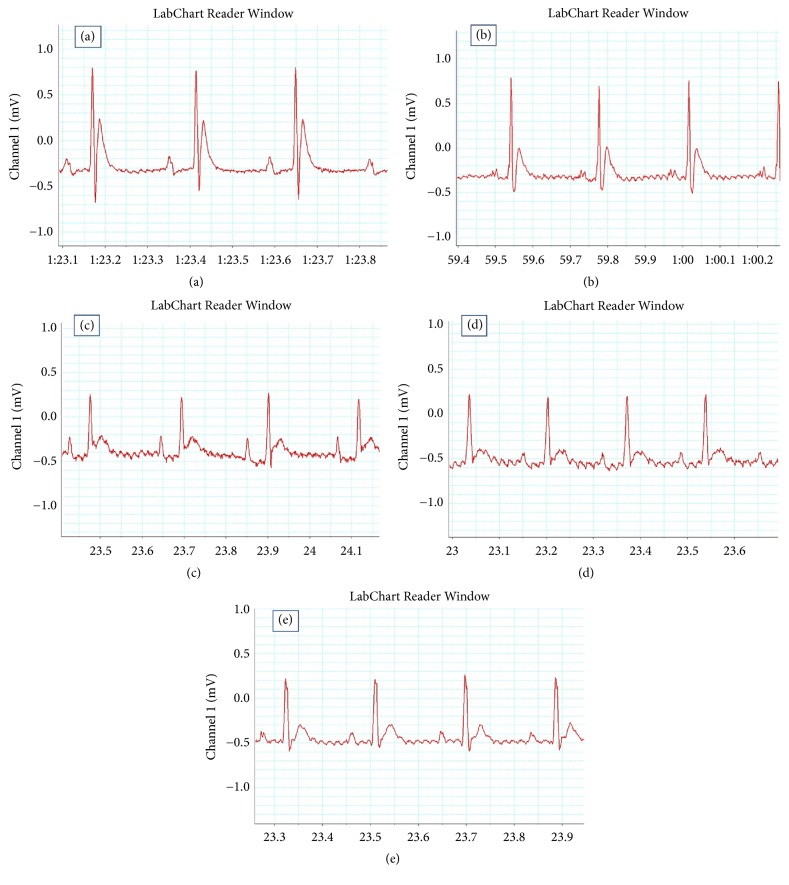
Effects of* A. fragrantissima* on Adr-induced changes in ECG tracing pattern. (a) Control group, (b)* A. fragrantissima* 800 group, (c) Adr group, (d)* A. fragrantissima* 400 + Adr group, and (e)* A. fragrantissima* 800 + Adr group.

The ECG of the control and* A. fragrantissima* 800 groups presented a normal-pattern (Figures 1(a) and 1(b)). Injection of rats with Adr (10 mg/kg) induced remarkable ST-segment elevation (Figure 1(c)). Pretreatments of rats with* A. fragrantissima* extract at both 400 and 800 mg/kg ameliorated Adr-induced ST-segment elevation (Figures 1(d) and 1(e)). In addition, injection of rats with Adr (10 mg/kg) significantly (p ≤ 0.05) increased heart rate and QT interval compared to the control group (Table 2). Pretreatments of rats with* A. fragrantissima* extract at both 400 and 800 mg/kg had no effect on Adr-induced increase in both heart rate and QT interval (Table 2).

### 3.3. Effects of* A. fragrantissima* on Serum CK-MB and LDH Measured in Adr-Induced Cardiotoxicity in Rats

Injection of rats with Adr (10 mg/kg) significantly elevated (p ≤ 0.05) both serum CK-MB and LDH levels compared to the control group. Pretreatments of rats with* A. fragrantissima* extract at both 400 and 800 mg/kg significantly reduced (p ≤ 0.05) both serum CK-MB and LDH levels compared to the Adr group. However, rats pretreated with 800 mg/kg* A. fragrantissima* showed significantly lower (p ≤ 0.05) serum CK-MB and LDH levels compared to 400 mg/kg* A. fragrantissima* + Adr group (Table 3).

### 3.4. Effects of* A. fragrantissima* on Heart Tissue Concentration of TBARS, GSH-PX, and GSH Measured in Adr-Induced Cardiotoxicity in Rat

Injection of rats with Adr (10 mg/kg) significantly elevated (p ≤ 0.05) heart tissue TBARS concentration compared to the control group. Pretreatments of rats with* A. fragrantissima* extract at both 400 and 800 mg/kg significantly reduced (p ≤ 0.05) heart tissue TBARS concentration compared to the Adr group. However, rats pretreated with 800 mg/kg* A. fragrantissima* showed significantly lower (p ≤ 0.05) heart tissue TBARS concentration compared to 400 mg/kg* A. fragrantissima* + Adr group (Table 4).

Injection of rats with Adr (10 mg/kg) significantly reduced (p ≤ 0.05) both heart tissue GSH-PX and GSH concentration compared to the control group. Pretreatments of rats with* A. fragrantissima *extract at both 400 and 800 mg/kg significantly elevated (p ≤ 0.05) both heart tissue GSH-PX and GSH concentration compared to the Adr group. However, rats pretreated with 800 mg/kg* A. fragrantissima* showed significantly higher (p ≤ 0.05) both heart tissue GSH-PX and GSH concentration compared to 400 mg/kg* A. fragrantissima* + Adr group (Table 4).

### 3.5. Effects of* A. fragrantissima* on Serum TNF-*α*, IL-1*β*, and IL-6 Measured in Adr-Induced Cardiotoxicity in Rats

**Figure 2:**
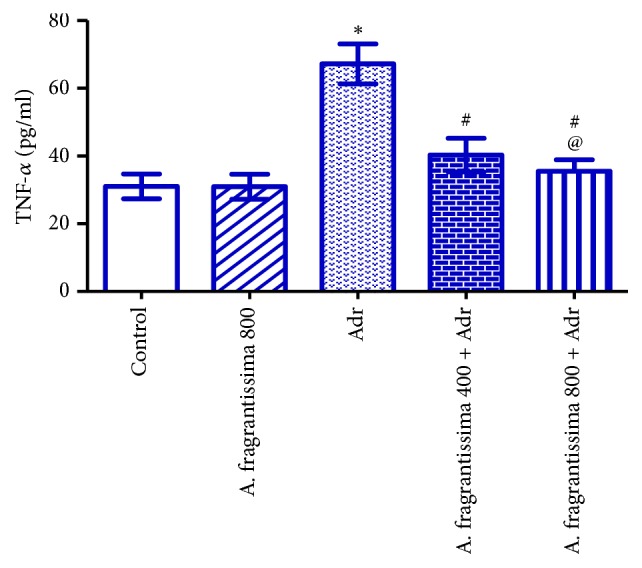
Effects of* A. fragrantissima* on serum concentration of TNF-*α* measured in control and Adr-induced cardiotoxic rats. Data are represented as mean ± SE (n = 10).  ^*∗*^Significant versus control group, ^#^significant versus Adr group, and ^@^significant between low and high dose group. p ≤ 0.05.

**Figure 3:**
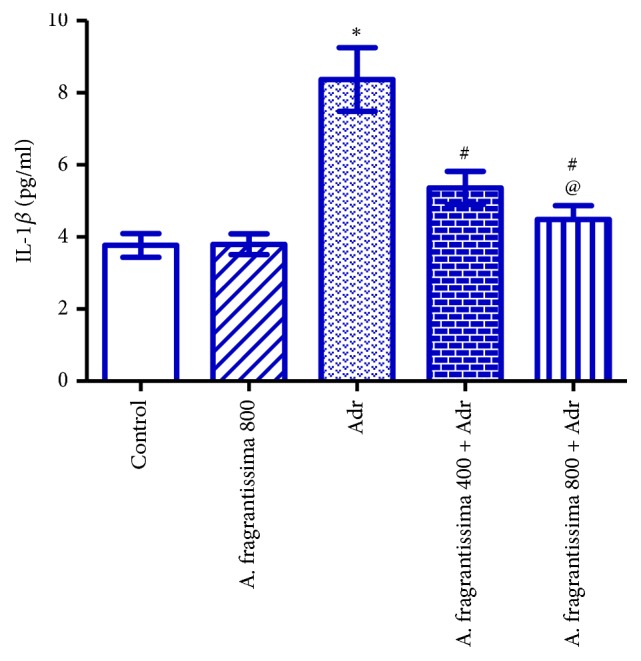
Effects of* A. fragrantissima* on serum concentration of IL-1*β* measured in control and Adr-induced cardiotoxic rats. Data are represented as mean ± SE (n = 10).  ^*∗*^Significant versus control group, ^#^significant versus Adr group, and ^@^significant between low and high dose group. p ≤ 0.05.

**Figure 4:**
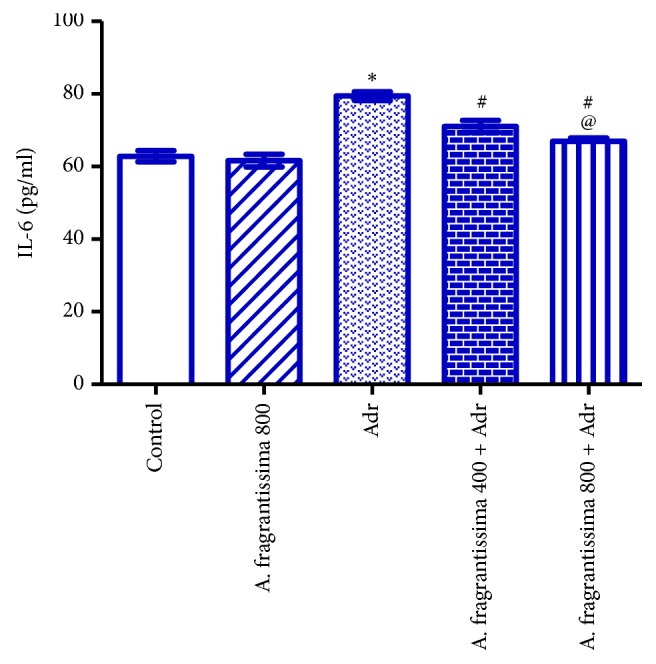
Effects of* A. fragrantissima* on serum concentration of IL-6 measured in control and Adr-induced cardiotoxic rats. Data are represented as mean ± SE (n = 10).  ^*∗*^Significant versus control group, ^#^significant versus Adr group, and ^@^significant between low and high dose group. p ≤ 0.05.

Injection of rats with Adr (10 mg/kg) significantly elevated (p ≤ 0.05) serum TNF-*α*, IL-1*β*, and IL-6 levels compared to the control group. Pretreatments of rats with* A. fragrantissima* extract at both 400 and 800 mg/kg significantly reduced (p ≤ 0.05) serum TNF-*α*, IL-1*β*, and IL-6 levels compared to the Adr group. However, rats pretreated with 800 mg/kg* A. fragrantissima* showed significantly lower (p ≤ 0.05) serum TNF-*α*, IL-1*β*, and IL-6 levels compared to 400 mg/kg* A. fragrantissima* + Adr group (Figures 2, 3, and 4).

### 3.6. Effects of* A. fragrantissima* on the Heart Tissue Histopathological Changes Detected by H & E Staining in Adr-Induced Cardiotoxicity in Rat

**Figure 5:**
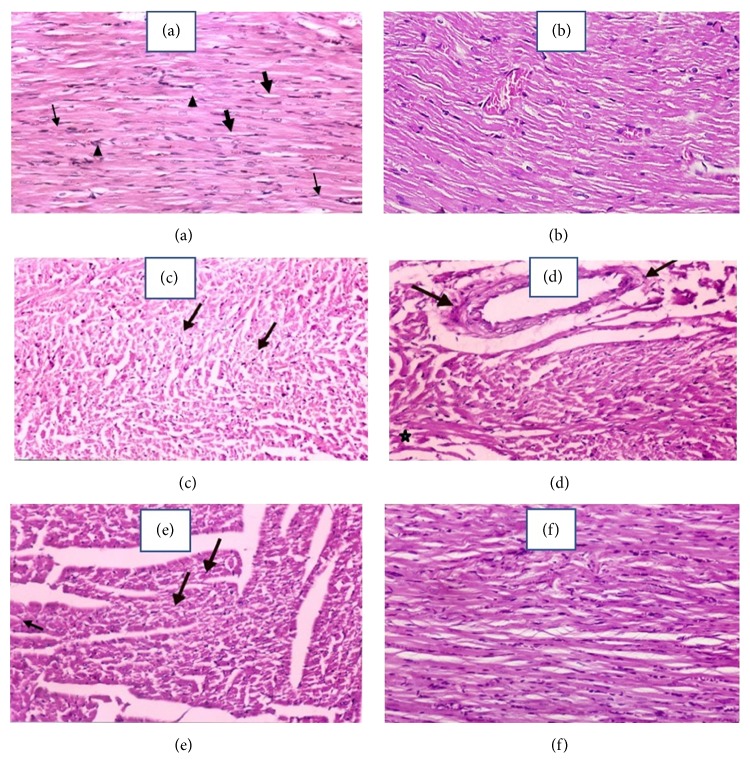
Effects of* A. fragrantissima* on the heart tissue histopathological changes detected by H & E staining in Adr-induced cardiotoxicity in rat (low power magnification ×200). Photo (a) represents control group, cardiac muscle showing branching, anastomosing, and striated muscle fibers with central oval vesicular nuclei (▸) and acidophilic cytoplasm. Notice the interstitial blood capillaries (→) and spindle-shaped connective tissue cells (thick arrow). Photo (b) represents* A. fragrantissima* 800 group. Photo (c) represents Adr group, cardiac muscle showing pale cytoplasm with pyknotic nuclei (thick arrows). Focal areas of widely separated cardiac muscle fibers ranging from degenerated cytoplasm up to necrosis. Photo (d) represents Adr group, cardiac muscle showing the perivascular edema around a coronary blood vessel (thick arrows) together with marked disorganization of the thin degenerated fibers (*∗*). Notice the scattered pyknotic nuclei in the damaged fibers. Photo (e) represents* A. fragrantissima* 400 + Adr group, showing necrosis of focal areas of degenerated cardiac muscle fiber (thick arrows). Notice the dark small pyknotic nuclei of muscle fibers with abnormal staining (arrow head). Photo (f) represents* A. fragrantissima* 800 + Adr group, showing the control appearance of the branching, anastomosing, and striated cardiac muscle.

**Figure 6:**
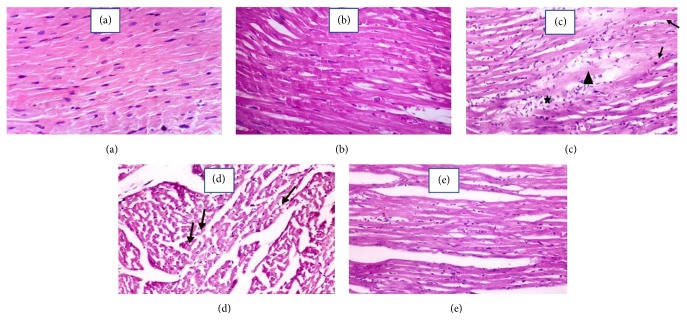
Effects of* A. fragrantissima* on the heart tissue histopathological changes detected by H & E staining in control and Adr-induced cardiotoxicity in rat (high-power magnification ×400). Photo (a) represents the cardiac muscle of control group. Photo (b) represents the cardiac muscle of* A. fragrantissima* 800 group, showing nearly normal cardiac muscle. Photo (c) represents the cardiac muscle of Adr group, showing focal disruption of some fibers (→) with pale acidophilic sarcoplasm. Notice the marked focal aggregation of inflammatory mononuclear cells (*∗*), interstitial fibroblasts (arrow head), and edema in between the fibers. Photo (d) represents the cardiac muscle of* A. fragrantissima* 400 + Adr group, showing the widely separated degenerated myocardial fibers with pale homogenous cytoplasm (thick arrows). Notice focal cytoplasmic vacuolization of some cardiac muscle fibers (→). Photo (e) represents the cardiac muscle of* A. fragrantissima* 800 + Adr group, showing the normal branched appearance of the cardiac muscle fibers.

Figures 5(a) and 6(a) showed control heart with normal histology. Figures 5(b) and 6(b) showed* A. fragrantissima* 800 heart with nearly normal histology. Injection of Adr (10 mg/kg) resulted in focal areas of necrosis, perivascular edema around a coronary blood vessel, marked disorganization of the thin degenerated fibers (Figures 5(c) and 5(d)). The high-power examination revealed a marked focal aggregation of inflammatory mononuclear cells in Adr-treated rats (Figure 6(c)). The heart tissue of the rats treated with* A. fragrantissima* extract at 400 mg/kg before Adr showed focal areas of necrosis (Figures 5(e) and 6(d)). On the other hand, pretreatment of Adr injected rats with* A. fragrantissima* extract at 800 mg/kg resulted in nearly complete protection from Adr-induced histopathological changes (Figures 5(f) and 6(e)).

## 4. Discussion

Adriamycin is one of the most effective chemotherapeutic agents which is widely used in the treatment of many tumors. The clinical uses of Adr are hampered by many associated toxicities, such as cardiac, renal, and pulmonary toxicity [29]. Adr administration may cause acute cardiac toxicity, which ranges from both ventricular and atrial arrhythmia to congestive heart failure [30, 31]. This study aimed to investigate the possible protective effect of* A. fragrantissima *extract against Adr-induced acute cardiotoxicity in rats.

The results of this study revealed that pretreatment of Adr injected rats with* A. fragrantissima* extract protected the rats against Adr-induced cardiotoxicity as manifested by the decreased cardiac enzymes, LDH and CK-MB. In addition, the histopathological study of the heart muscle revealed that* A. fragrantissima *extract improved Adr-induced histopathological and ultrastructural damage.

In this study, we found that Adr moderately prolonged QT interval, increased heart rate, and provoked ST-segment elevation. These results are consistent with many previous findings which suggested QT interval, an indicator of ventricular function to be prolonged during Adr toxicity via an oxidative stress mechanism [29, 32]. In addition, Adr-increased ROS generation causes ST-segment elevation which is an important marker of ischemia [33]. Pretreatment of Adr injected rats with* A. fragrantissima* extract moderately ameliorated Adr-induced ST-segment elevation. However,* A. fragrantissima* extract failed to reverse Adr-induce tachycardia and QT prolongation.

The imbalance between free radicals and antioxidant enzymes is considered the most important mechanism behind Adr-developed cardiac toxicity [30, 34]. The present results demonstrated that, following Adr injection, cardiac levels of TBARS were moderately increased, and that the levels of the cardiac antioxidant GSH-PX and GSH were moderately reduced. This information obviously points a case of distinct oxidative stress. These data are in consistent with the recent results of Benzer* et al*. [35].* A. fragrantissima* extract moderately decreased the Adr-associated elevation of heart tissue content of TBARS, which is the product of Adr-induced lipid peroxidation. Furthermore,* A. fragrantissima* extract moderately increased the heart tissue content of GSH-PX and GSH, which are important antioxidant defense of the heart. Similarly, a previous study has shown that Achilloid A, a sesquiterpene lactone, one of the most biologically active constituents of* A. fragrantissima*, could resist the oxidative stress caused by H_2_O_2_ in astrocytes via scavenging of ROS and blocking of H_2_O_2_-induced mitogen-activated protein kinase (MAPK) pathway [19]. This study also showed high levels of phenolic and flavonoid compounds in the* A. fragrantissima* extract. Like our results, the methanolic extract of* A. fragrantissima* had a large amount of phenolic and flavonoid compounds and exerted a strong antioxidant activity in the diphenylpicrylhydrazyl (DPPH) free radical scavenging assay [20, 36]. The phenolic compounds display great ROS scavenging efficacy, via their reactivity as hydrogen or electron-donating substances, and metal ion chelating attributes [37, 38].

Several mechanistic pathways are probable to be implicated in Adr-induced cardiotoxicity including increased inflammatory reactions within the myocardium and promoted the release of proinflammatory cytokines including TNF-*α* and IL-1*β* [39]. In addition, novel clinical proof props the possible impact of IL-8 in atherosclerosis, both as a sign and as a probable medicinal goal. In the area of interventional cardiology, it was proposed that high serum concentration of IL-8 after percutaneous coronary intervention could prognosticate the evolution of cardiac failure in humans with acute myocardial infarction [40, 41]. NFkB signaling is a converging point for controlling downstream signaling cascades that include TNF-*α*, IL-1*β*, IL-6, IL-8, and transcription of other inflammatory genes [42].

Beyond their antioxidant effects,* A. fragrantissima* extract has frequently been shown to possess various anti-inflammatory and immunomodulatory effects [19]. The present study demonstrated that pretreatment with* A. fragrantissima* caused significant reductions in serum TNF-*α*, IL-1*β*, and IL-6 levels, suggesting a reliable cytoprotective action of* A. fragrantissima* against Adr-mediated release of proinflammatory mediators. Similarly,* A. fragrantissima* extract inhibited lipopolysaccharide (LPS)-induced expression of TNF-*α* and IL-1*β* and downregulated ROS production from primary cultures of activated microglial cells [19]. The mechanism by which* A. fragrantissima* reduced TNF-*α*, IL-1*β*, and IL-6 concentrations following Adr injection has not been elucidated in this work. However, the anti-inflammatory effect of* A. fragrantissima* extract may result from its antioxidant active constituents that can cross the cell membranes and scavenge the ROS intracellularly [19]. Therefore, the inhibitory effect of the* A. fragrantissima* extract on the expression of TNF-*α* and IL-1*β* might be attributed to inhibition of NFkB activation or to other signaling events leading to the production of proinflammatory molecules in myocytes such as protein kinase C or p38 MAPK [43].

## 5. Conclusion

The results of this study revealed that* A. fragrantissima* extract ameliorates Adr-induced cardiotoxicity as it ameliorated ST-segment elevation and reduced LDH and CK-MB via an antioxidant (decreased TBARS and increased GSH and GSH-PX) and anti-inflammatory (decreased TNF-*α*, IL-1*β*, and IL-6) mechanisms. Further studies are recommended to elucidate the specified active constituents of* A. fragrantissima* which are lies beneath its cardioprotective effect through the antioxidant and anti-inflammatory actions.

## Figures and Tables

**Table 1:** Phytochemical analysis of *A. fragrantissima* extract.

Phytochemical compounds	Inference
Flavonoids	+++
Phenolic	+++
Glycosides	+++
Terpenoids	++
Tannins	++
Alkaloids	++
Lignans	+++
Steroids	++
Terpenes	+++
Saponins	+
Anthraquinone	+
Triterpenes	-
Resins	-

The intensity of compounds: - absence, + minute, ++ small, and +++ large.

**Table 2:** Effects of *A. fragrantissima* on control and Adr-induced changes in EGG parameters.

Experimental Groups	Heart Rate	QT interval
	(beat/min)	(s)
Control	251 ± 13	0.052 ± 0.003
*A. fragrantissima *800	255 ± 15	0.05 ± 0.001
Adr	319 ± 19 ^a^	0.071 ± 0.004 ^a^
*A. fragrantissima *400 + Adr	328 ± 10	0.069 ± 0.005
*A. fragrantissima *800 + Adr	316 ± 12	0.071 ± 0.002

Data are represented as mean ± SE (n = 10).  ^a^Significant versus control group. p ≤ 0.05.

**Table 3:** Effects of *A. fragrantissima* on serum CK-MB and LDH measured in control and Adr-induced cardiotoxic rats.

Experimental Groups	CK-MB	LDH
	(U/L)	(U/L)
Control	257.27 ± 10.87	249.29 ± 11.74
*A. fragrantissima *800	253.4 ± 9.95	247.96 ± 12.10
Adr	438.52 ± 15.87 ^a^	529.89 ± 14.72 ^a^
*A. fragrantissima *400 + Adr	306.01 ± 10.75 ^b^	316.28 ± 14.91 ^b^
*A. fragrantissima *800 + Adr	276.40 ± 8.09 ^b,c^	271.78 ± 8.10 ^b,c^

Data are represented as mean ± SE (n = 10).  ^a^Significant versus control group, ^b^significant versus Adr group, and ^C^significant between low and high dose group. p ≤ 0.05.

**Table 4:** Effects of *A. fragrantissima* on heart tissue concentration of TBARS, GSH-PX, and GSH measured in control and Adr-induced cardiotoxic rats.

Experimental Groups	TBARS	GSH-PX	GSH
	(nmol/g tissue)	(nmol/mg tissue)	(nmol/g tissue)
Control	4.76 ± 0.49	27.89 ± 1.51	4.34 ± 0.08
*A. fragrantissima *800	4.51 ± 0.46	28.12 ± 1.87	4.75 ± 0.15
Adr	9.16 ± 0.84 ^a^	12.73 ± 1.66 ^a^	1.78 ± 0.06 ^a^
*A. fragrantissima *400 + Adr	6.29 ± 0.53 ^b^	23.61 ± 1.58 ^b^	3.61 ± 0.12 ^b^
*A. fragrantissima *800 + Adr	5.18 ± 0.44 ^b,c^	27.17 ± 1.44 ^b,c^	4.14 ± 0.09 ^b,c^

Data are represented as mean ± SE (n = 10).  ^a^Significant versus control group,  ^b^significant versus Adr group, and  ^C^significant between low and high dose group. p ≤ 0.05.

## Data Availability

The data and materials supporting the conclusions of this article are included within the article.

## References

[1] Fernandez-Chas M., Curtis M. J., Niederer S. A. (2018). Mechanism of doxorubicin cardiotoxicity evaluated by integrating multiple molecular effects into a biophysical model. *British Journal of Pharmacology*.

[2] Mele D., Tocchetti C. G., Pagliaro P. (2016). Pathophysiology of anthracycline cardiotoxicity. *Journal of Cardiovascular Medicine*.

[3] Maurea N., Coppola C., Piscopo G. (2016). Pathophysiology of cardiotoxicity from target therapy and angiogenesis inhibitors. *Journal of Cardiovascular Medicine*.

[4] Šimůnek T., Štěrba M., Popelová O., Adamcová M., Hrdina R., Gerši V. (2009). Anthracycline-induced cardiotoxicity: overview of studies examining the roles of oxidative stress and free cellular iron. *Pharmacological Reports*.

[5] Wu K., Schwartz S. J., Platz E. A. (2003). Variations in plasma lycopene and specific isomers over time in a cohort of U.S. men. *Journal of Nutrition*.

[6] El-Aziz T. A. A., Mohamed R. H., Pasha H. F., Abdel-Aziz H. R. (2012). Catechin protects against oxidative stress and inflammatory-mediated cardiotoxicity in adriamycin-treated rats. *Clinical and Experimental Medicine*.

[7] Guo R., Wu K., Chen J. (2013). Exogenous hydrogen sulfide protects against doxorubicin-induced inflammation and cytotoxicity by inhibiting p38MAPK/NF*κ*B pathway in H9c2 cardiac cells. *Cellular Physiology and Biochemistry*.

[8] Zhu J., Zhang J., Zhang L. (2011). Interleukin-1 signaling mediates acute doxorubicin-induced cardiotoxicity. *Biomedicine & Pharmacotherapy*.

[9] Hosseini A., Bakhtiari E., Mousavi S. H. (2017). Protective effect of hibiscus sabdariffa on doxorubicin-induced cytotoxicity in H9c2 cardiomyoblast cells. *Iranian Journal of Pharmaceutical Research*.

[10] Capasso I., Esposito E., Maurea N. (2013). Combination of inositol and alpha lipoic acid in metabolic syndrome-affected women: a randomized placebo-controlled trial. *Trials*.

[11] Møller P., Loft S., Lundby C., Olsen N. V. (2001). Acute hypoxia and hypoxic exercise induce DNA strand breaks and oxidative DNA damage in humans. *The FASEB Journal*.

[12] Nakamura K., Fushimi K., Kouchi H. (1998). Inhibitory effects of antioxidants on neonatal rat cardiac myocyte hypertrophy induced by tumor necrosis factor-alpha and angiotensin II. *Circulation*.

[13] Shabana M. M., Mirhom Y. W., Genenah A. A., Aboutabl E. A., Amer H. A. (1990). Study into wild Egyptian plants of potential medicinal activity. Ninth communication: hypoglycaemic activity of some selected plants in normal fasting and alloxanised rats. *Archiv für Experimentelle Veterinärmedizin*.

[14] Mustafa E. H., Abu Zarga M., Abdalla S. (1992). Effects of cirsiliol, a flavone isolated from Achillea fragrantissima, on rat isolated ileum. *General Pharmacology: The Vascular System*.

[15] Yaniv Z., Dafni A., Friedman J., Palevitch D. (1987). Plants used for the treatment of diabetes in Israel. *Journal of Ethnopharmacology*.

[16] Hamdan I. I., Afifi F. U. (2004). Studies on the in vitro and in vivo hypoglycemic activities of some medicinal plants used in treatment of diabetes in Jordanian traditional medicine. *Journal of Ethnopharmacology*.

[17] Ezzat S. M., Salama M. M. (2014). A new *α*-glucosidase inhibitor from Achillea fragrantissima (Forssk.) Sch. Bip. growing in Egypt. *Natural Product Research (Formerly Natural Product Letters)*.

[18] Elmann A., Telerman A., Mordechay S. (2015). Downregulation of microglial activation by achillolide A. *Planta Medica*.

[19] Mohamed A. A., Ali S. I., El-Baz F. K., El-Senousy W. M. (2015). New insights into antioxidant and antiviral activities of two wild medicinal plants: achillea fragrantissima and Nitraria Retusa. *International Journal of Pharma and Bio Sciences*.

[20] Merfort I. (2011). Perspectives on sesquiterpene lactones in inflammation and cancer. *Current Drug Targets*.

[21] Kim S.-K., Cho S.-B., Moon H.-I. (2010). Neuroprotective effects of a sesquiterpene lactone and flavanones from Paulownia tomentosa Steud. against glutamate-induced neurotoxicity in primary cultured rat cortical cells. *Phytotherapy Research*.

[22] Choi E. M., Kim G.-H., Lee Y. S. (2009). Protective effects of dehydrocostus lactone against hydrogen peroxide-induced dysfunction and oxidative stress in osteoblastic MC3T3-E1 cells. *Toxicology in Vitro*.

[23] Gach K., Długosz A., Janecka A. (2015). The role of oxidative stress in anticancer activity of sesquiterpene lactones. *Naunyn-Schmiedeberg's Archives of Pharmacology*.

[24] Alenad A. M., Al-Jaber N. A., Krishnaswamy S., Yakout S. M., Al-Daghri M. N., Alokail M. S. (2013). Achillea fragrantissima extract exerts its anticancer effect via induction of differentiation, cell cycle arrest and apoptosis in chronic myeloid leukemia (CML) cell line K562. *Journal of Medicinal Plants Research*.

[25] Harborne A. J. (1998). *Phytochemical Methods: A Guide to Modern Techniques of Plant Analysis*.

[26] El-Shitany N. A., El-Haggar S., El-desoky K. (2008). Silymarin prevents adriamycin-induced cardiotoxicity and nephrotoxicity in rats. *Food and Chemical Toxicology*.

[27] Hosseini M., Harandizadeh F., Niazamand S., Soukhtanloo M., Mahmoudabady M. (2013). Antioxidant effect of Achillea wilhelmsii extract on pentylenetetrazole (seizure model)-induced oxidative brain damage in Wistar rats. *Indian Journal of Physiology and Pharmacology*.

[28] Azhar A., El-Bassossy H. M. (2014). Pentoxifylline alleviates cardiac ischemia and dysfunction following experimental angina in insulin resistance. *PLoS ONE*.

[29] Khattab H. A. H., El-Shitany N. A., Al-Lily A. K. S. (2016). Effect of roselle (Hibiscus sabdariffa) against adriamycin induced-cardiotoxicity in male rats. *The International Journal of Pharmaceutical Research and Allied Sciences*.

[30] Asensio-López M. C., Soler F., Pascual-Figal D., Fernández-Belda F., Lax A. (2017). Doxorubicin-induced oxidative stress: the protective effect of nicorandil on HL-1 cardiomyocytes. *PLoS ONE*.

[31] Jain D., Ahmad T., Cairo M., Aronow W. (2017). Cardiotoxicity of cancer chemotherapy: identification, prevention and treatment. *Annals of Translational Medicine*.

[32] Koti B. C., Nagathan S., Vishwanathswamy A., Gadad P. C., Thippeswamy A. (2013). Cardioprotective effect of Vedic Guard against doxorubicin-induced cardiotoxicity in rats: a biochemical, electrocardiographic, and histopathological study. *Pharmacognosy Magazine*.

[33] El-Sayed E. M., Abd El-azeem A. S., Afify A. A., Shabana M. H., Ahmed H. H. (2011). Cardioprotective effects of Curcuma longa L. extracts against doxorubicin-induced cardiotoxicity in rats. *Journal of Medicinal Plants Research*.

[34] Duggan S. T., Keating G. M. (2011). Pegylated Liposomal doxorubicin. *Drugs*.

[35] Benzer F., Kandemir F. M., Ozkaraca M., Kucukler S., Caglayan C. (2018). Curcumin ameliorates doxorubicin-induced cardiotoxicity by abrogation of inflammation, apoptosis, oxidative DNA damage, and protein oxidation in rats. *Journal of Biochemical and Molecular Toxicology*.

[36] Shahat A. A., Alsaid M. S. (2015). Antioxidant capacity and polyphenolic content of seven Saudi Arabian medicinal herbs traditionally used in Saudi Arabia. *Indian Journal of Traditional Knowledge*.

[37] Rice-Evans C. A., Miller N. J., Paganga G. (1996). Structure-antioxidant activity relationships of flavonoids and phenolic acids. *Free Radical Biology & Medicine*.

[38] Lu Y., Foo L. Y. (2001). Antioxidant activities of polyphenols from sage (*Salvia officinalis*). *Food Chemistry*.

[39] Shaker R. A., Abboud S. H., Assad H. C., Hadi N. (2018). Enoxaparin attenuates doxorubicin induced cardiotoxicity in rats via interfering with oxidative stress, inflammation and apoptosis. *BMC Pharmacology & Toxicology*.

[40] Quagliariello V., Vecchione R., Coppola C. (2018). Cardioprotective effects of nanoemulsions loaded with anti-inflammatory nutraceuticals against doxorubicin-induced cardiotoxicity. *Nutrients*.

[41] Apostolakis S., Vogiatzi K., Amanatidou V., Spandidos D. A. (2009). Interleukin 8 and cardiovascular disease. *Cardiovascular Research*.

[42] Vyas D., Laput G., Vyas A. K. (2014). Chemotherapy-enhanced inflammation may lead to the failure of therapy and metastasis. *OncoTargets and Therapy*.

[43] Elmann A., Mordechay S., Erlank H., Telerman A., Rindner M., Ofir R. (2011). Anti-Neuroinflammatory effects of the extract of Achillea fragrantissima. *BMC Complementary and Alternative Medicine*.

